# Active Chromatin Marks Are Retained on X Chromosomes Lacking Gene or Repeat Silencing Despite *XIST/Xist* Expression in Somatic Cell Hybrids

**DOI:** 10.1371/journal.pone.0010787

**Published:** 2010-05-24

**Authors:** Nancy P. Thorogood, Carolyn J. Brown

**Affiliations:** Department of Medical Genetics, University of British Columbia, Vancouver, Canada; Duke University, United States of America

## Abstract

**Background:**

X-chromosome inactivation occurs early in mammalian development and results in the inactive X chromosome acquiring numerous hallmarks of heterochromatin. While XIST is a key player in the inactivation process, the method of action of this ncRNA is yet to be determined.

**Methodology/Principal Findings:**

To assess which features of heterochromatin may be directly recruited by the expression and localization of the XIST RNA we have analyzed a mouse/human somatic cell hybrid in which expression of human and mouse *XIST/Xist* has been induced from the active X by demethylation. Such hybrids had previously been demonstrated to disconnect *XIST/Xist* expression from gene silencing and we confirm maintenance of X-linked gene expression, even close to the *Xist* locus, despite the localized expression of mouse Xist.

**Conclusions/Significance:**

Loss of the active chromatin marks H3 acetylation and H3 lysine 4 methylation was not observed upon *XIST/Xist* expression, nor was there a gain of DNA methylation; thus these marks of facultative heterochromatin are not solely dependent upon *Xist* expression. Cot-1 holes, regions of depleted RNA hybridization with a Cot-1 probe, were observed upon *Xist* expression; however, these were at reduced frequency and intensity in these somatic cells. Domains of human Cot-1 transcription were observed corresponding to the human chromosomes in the somatic cell hybrids. The Cot-1 domain of the X was not reduced with the expression of XIST, which fails to localize to the human X chromosome in a mouse somatic cell background. The human inactive X in a mouse/human hybrid cell also shows delocalized XIST expression and an ongoing Cot-1 domain, despite X-linked gene silencing. These results are consistent with recent reports separating Cot-1 silencing from genic silencing, but also demonstrate repetitive element expression from an otherwise silent X chromosome in these hybrid cells.

## Introduction

X-chromosome inactivation achieves dosage compensation of X-linked genes between mammalian XX females and XY males. The non-coding RNA XIST, which is expressed from the inactive X (Xi), but not the active X (Xa), is a critical initiator of X inactivation in eutheria. Inactivation occurs early in development, thus much of our knowledge regarding the initial events has been derived from studies utilizing mouse embryos or differentiating mouse ES cells containing *Xist* transgenes or mutations (reviewed in [1]). During X-chromosome inactivation, the Xist RNA coats the Xi, initiating gene silencing and the establishment of a heterochromatic state. This process leads to identifiable differences between the Xa and the Xi, some of which may be directly recruited by XIST/Xist, while others are likely indirect and due to the silenced nature of the chromatin. The chromatin of the Xi is distinguished by decreased active histone modifications, such as H3 and H4 acetylation, as well as increased inactive modifications, including the association of a histone variant, macroH2A, and DNA methylation at the CpG islands of gene promoters (reviewed in [1]). Furthermore, the Xi is associated with the formation of a transcriptionally silenced domain that can be visualized by lack of RNA hybridization with a probe for the Cot-1 fraction of genomic DNA and is thus referred to as a Cot-1 hole [2,3,4].

Model systems in which *Xist* expression is disconnected from silencing provide a tool to determine what chromatin changes are directly recruited by the Xist RNA. Induction of mouse *Xist* in somatic cells from an exogenous promoter was unable to induce silencing and allowed the definition of a limited developmental window in early ES cell differentiation during which silencing can be established [5]. The analysis of deletion constructs of an inducible *Xist* cDNA identified a transgene lacking the conserved 5′A-repeat region (Xist-delSX) that was able to produce localized Xist RNA but was not able to induce transcriptional silencing. The expression of Xist-delSX in differentiating mouse ES cells demonstrated that despite a lack of Xist-induced silencing, a variety of downstream repressive epigenetic marks can be recruited following *Xist* expression [2], [6], [7], [8], [9], [10], [11].

To address the role of XIST/Xist in establishing chromatin changes on the Xi, we have expanded on previous studies of mouse/human somatic cell hybrids where *XIST/Xist* expression induced by treatment with the DNA demethylating agent 5-azacytidine (5-aza) did not result in *de novo* transcriptional silencing of the Xa [12]. We now report a mouse/human somatic cell hybrid that stably expressed both human and mouse *XIST/Xist* from the human and mouse X chromosomes. The mouse Xist RNA localized to the mouse X, however, the human XIST RNA did not localize to the human X. As silencing of genes closest to the *Xist* locus has been shown to occur first in early development [4], [13], we have revisited the question of silencing and show that activation of *Xist* from the endogenous locus in somatic cells is incapable of silencing even genes situated close to the *Xist* locus. This confirmed the utility of these cells as a model system in which silencing is disconnected from *XIST/Xist* expression, with or without localization of the ncRNA.

We demonstrate that the *XIST/Xist*-expressing chromosome retains characteristics of an Xa, including DNA hypomethylation and both H3K4 dimethylation and H3 acetylation at promoter regions. These hybrid cells show very limited ability to form a Cot-1 hole corresponding to the mouse X chromosome, but demonstrate a domain of hybridization to human Cot-1 at human chromosomes in a mouse background. This Cot-1 hybridizing domain was also observed when XIST was induced and at human Xi's, demonstrating ongoing X-specific repetitive element expression in these hybrid cells in the absence of localized XIST but in the presence of genic silencing.

## Results

### XIST/Xist are Induced by Demethylation but Show Species Difference in Localization

**Figure 1:**
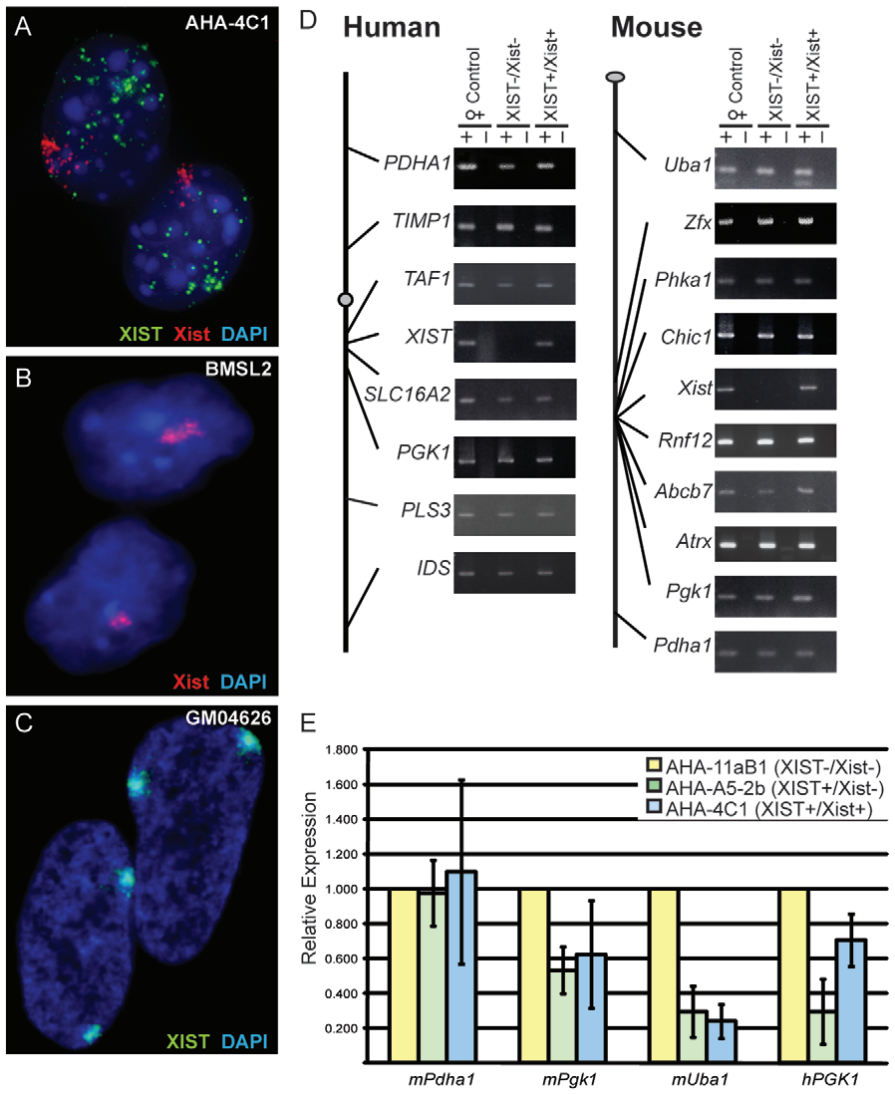
X-linked gene expression in somatic cell hybrids before and after expression of *XIST/Xist*. (A–C) Shows the *XIST/Xist* expression in cell nuclei using RNA FISH probes for the human XIST RNA (green) and the mouse Xist RNA (red). (A) The XIST^+^/Xist^+^ (AHA-4C1) somatic cell hybrid; (B) The female mouse fibroblast control cell line, BMSL2; (C) The human female fibroblast line GM04626 (karyotype: 47,XXX). (D) Schematic diagram of both the human and mouse X chromosomes showing the location of the genes analyzed to the left of gel images of RT-PCR products of cDNA from the control female (GM7350 (human) and BMSL2 (mouse)); the hybrid not expressing AHA-11aB1 (XIST^−^/Xist^−^); and the derivative hybrid that expresses AHA-4C1 (XIST^+^/Xist^+^). (E) Quantitative RT-PCR of 4 X-linked genes. An intermediate hybrid AHA-A5-2b (XIST^+^/Xist^−^) was analyzed for expression along with the hybrid AHA-4C1 (XIST^+^/Xist^+^). The gene expression was normalized to Actin and expressed as a fold-change relative to the parent hybrid AHA-11aB1 (XIST^−^/Xist^−^). The error bars represent the standard deviation of three separate RNA isolations.

The mouse/human hybrid cell line AHA-11aB1 retains only the human Xa [14], and a subclone was previously described that stably expressed human *XIST* after three rounds of demethylation with 5aza (AHA-A5-2b) [15]. We have now subjected AHA-A5-2b to further rounds of demethylation to yield a new subclone designated AHA-4C1 that stably expresses both human *XIST* and mouse *Xist*. In these AHA-4C1 cells FISH analysis demonstrated that the human XIST RNA was not localized within the nucleus while mouse Xist RNA was localized (Figure 1A). 76% of cells had Xist localization and a diffuse XIST signal (n = 95), similar to numbers reported by Clemson *et al* when they analyzed a transiently-expressing hybrid [12]. Quantitative PCR analysis showed that mouse Xist was expressed equivalently in the hybrid cell line (AHA-4C1) and the female mouse cell line (BMSL2) used as a control (data not shown).

### Absence of X-linked Gene Silencing upon Induction of XIST/Xist

Previous studies have shown that both the human and mouse X chromosomes remained active upon induction of *XIST/Xist* expression [12], [15], [16]. Expression studies have shown that early in development silencing occurs in a gradient [4], [13], with genes closest to *Xist* showing the greatest degree of inactivation. Therefore, we directly assessed transcriptional status of human and mouse X-linked genes in the AHA-4C1 cells by RT-PCR, but focused in the *Xist* region (see Figure 1D and E). To test whether any silencing occurred closer to the *Xist* gene than previously examined, or after stable long-term *Xist* expression, we examined the expression of 16 genes, including *Chic1*, *Rnf12*, *Abcb7*, and *Atrx* which are within 2 Mb of *Xist*. Both mouse and human X chromosomes are shown schematically in Figure 1D with the placement of genes examined shown approximately to scale. Expression analysis with RT- PCR demonstrated that the expression of *XIST/Xist* did not result in transcriptional silencing of any of the 16 X-linked genes examined. A female human cell line (GM7350) and a female mouse cell line (BMSL2) were used as controls to show the normal transcription status of the genes tested. All genes, except *XIST/Xist*, were expressed in the cell line (AHA-11aB1) prior to *XIST/Xist* induction and continued to be expressed in the AHA-4C1 hybrid with the reactivated *XIST/Xist* loci.

To assess more subtle changes in gene expression we performed quantitative RT-PCR on three mouse and one human X-linked genes (Figure 1E). An additional cell line (AHA-A5-2b) was included as a control for the 5-aza treatment to determine whether any expression changes were a result of the demethylation process itself or *XIST/Xist* expression. The AHA-A5-2b cells had reactivated only the human *XIST* gene upon demethylation; therefore any changes in the expression status of mouse X-linked genes in this intermediate cell line should reflect the effect of the demethylation treatment and not *Xist* expression. The relative expression ratio was determined by dividing the expression level of the gene of interest by *Actin* levels, and then calculating the fold change in relative expression of the reactivated cell lines versus the parental hybrid. Three out of the four genes analyzed showed a change in expression; specifically, mouse *Pgk1*, *Uba1* and human *PGK1* had a decreased expression following the demethylation treatment in both the demethylated hybrid cell lines (AHA-A5-2b and AHA-4C1). The decrease in expression did not appear to be a result of the *XIST/Xist* expression since two mouse genes had this decrease in the intermediate cell line, AHA-A5-2b, that only expressed human *XIST* and not mouse *Xist*. Hence, the expression levels of *Pgk1*, *Uba1* and *PGK1* decreased as a result of the demethylation treatment rather than *Xist* expression.

We thus conclude that although the demethylation treatment caused fluctuations in gene expression, both the X chromosomes in these hybrid cells remained active despite *XIST/Xist* expression. These results validate our system as one in which *XIST/Xist* expression and the silencing of X-linked genes has been disconnected, thereby providing a model system in which X chromosome inactivation marks can be studied with respect to *XIST/Xist* expression in the absence of silencing.

### Diminished Mouse Cot-1 Hole Formation upon *Xist* Expression

**Figure 2:**
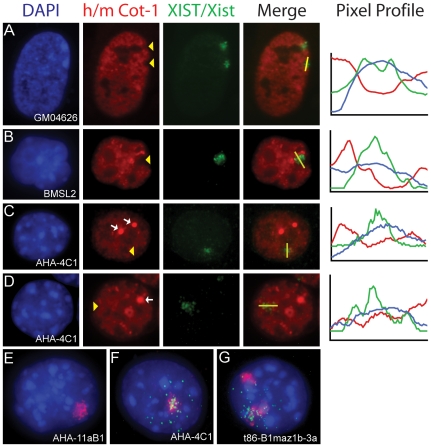
Analysis of Cot-1 hybridization in somatic cell hybrids expressing *Xist/XIST* by dual RNA FISH hybridization. Cot-1 RNA (mouse (m) or human (h)) signal is red, Xist/XIST RNA signal is green and DAPI is blue. (A–D) Lines were drawn in ImageJ (NIH) and pixel intensities of each signal was plotted and shown to the right of the images. Yellow arrow heads indicate where the Xist signal corresponds to reductions in the Cot-1 signal. (A) Human female cell line, GM04626, with two inactive X chromosomes and two hCot-1 holes corresponding to the two XIST RNA signals. (B) Mouse female cell line, BMSL2, showing one mCot-1 hole corresponding to the mouse Xist signal, this pattern was observed in 59% of cells (n = 100). (C) XIST^+^/Xist^+^ somatic cell hybrid, AHA-4C1, showing a mCot-1 hole corresponding to the mouse Xist signal, this pattern was observed in 7% of cells (n = 100). (D) AHA-4C1 without a mCot-1 hole. In panels C and D bright foci of mCot-1 staining are observed (white arrows). (E) hCot-1 RNA expression (red) in the XIST^−^/Xist^−^ hybrid cell, AHA-11aB1, containing only one human chromosome (the X chromosome). (F) hCot-1 RNA expression (red) in the XIST^+^/Xist^+^ hybrid cell, AHA-4C1, containing only one human chromosome (the X chromosome), and XIST RNA (green) drifting from the hCot-1 domain. (G) An Xi-hybrid cell line, t86-B1maz1b-3a, containing two human chromosome (X+15). Both human chromosomes form an hCot-1 transcriptional domain, one with the XIST signal drifting away.

Cot-1 DNA can be hybridized to RNA within the nucleus to identify heteronuclear RNA transcription [17]. Lack of Cot-1 staining has been observed at the location of the Xi and appropriately termed a ‘Cot-1 hole’. It has been suggested that this Cot-1 hole reflects X-chromosome silencing [17] and the formation of a repressive nuclear compartment [2]. We examined the presence of a Cot-1 hole using dual-RNA FISH with an *XIST/Xist* probe and the corresponding human or mouse Cot-1 probe (Figure 2). The commercially available Cot-1 DNA that was used to make the probe was species-specific, as mouse Cot-1 (mCot-1) showed no hybridization to human RNA under our hybridization conditions and vice-versa (data not shown).

In the hybrid cell line expressing *XIST/Xist* (AHA-4C1) we found that only 7% of the cells contained a mCot-1 hole corresponding to the localized Xist signal (n = 83), compared to 59% in the mouse control cell line (Figure 2B and C). To determine the signal intensity of mCot-1 RNA at the site of Xist localization we used line scans (ImageJ, NIH). The line scans confirmed that the increased signal intensity for the Xist RNA did not always correspond with a decrease in signal intensity for mCot-1 RNA (Figure 2D). Furthermore, not only were there fewer mCot-1 holes in the hybrid cells, but in those that were identified, the lack of mCot-1 signal was also less obvious as compared to the mouse control. The drastic reduction in the formation of Cot-1 holes showed that the expression of *Xist* in a somatic cell does not consistently promote the appearance of a Cot-1 hole.

The demethylated cells expressing *XIST/Xist* (AHA-4C1) also showed several mCot-1 bright regions, which varied in size and number from cell to cell (Figure 2C and D, white arrows). Since these cells were demethylated to induce *XIST/Xist* expression, we analyzed an Xi hybrid cell (t11-az-10) that expressed *XIST* before it was demethylated [18] and also observed the mCot-1 bright regions (data not shown). The presence of Cot-1 foci in both these demethylated cell lines suggests that their appearance is a result of the demethylation treatment and not *Xist* induction.

### Human Cot-1 ‘Domains’ are not Reduced by Dispersed *XIST* Expression

When we assessed the hCot-1 hybridization in the hybrid cell line expressing *XIST/Xist* (AHA-4C1) with a dispersed human XIST signal, one definitive hCot-1 RNA domain per cell was identified (Figure 2E and F). We used the Cot-1 domain to explore hybrid cell lines that contained additional human chromosomes. We found there was an equivalent increase in the number of hCot-1 RNA domains that corresponded to the number of human chromosomes (Figure 2G and data not shown). Since Cot-1 RNA hybridization represents heteronuclear RNA transcription, this observation suggests that in human-mouse fibroblast hybrid cells, human chromosomes form a transcriptional domain as visualized by hCot-1 hybridization.

To examine the effect of gene silencing on the transcriptional domain we examined hCot-1 in a hybrid cell that contained an Xi (t86-B1maz1-3a) [14]. In this particular cell line, the only human chromosomes within the mouse cell are the Xi and chromosome 15. If the Xi silenced hnRNA expression, as generally observed with the establishment of a Cot-1 hole, then only one hCot-1 domain would be observed corresponding to the human chromosome 15. However this was not the case, two hCot-1 domains were observed, one of which corresponded to the origin of the XIST signal (Figure 2G). This suggests that the repetitive DNA elements contained within the hCot-1 fraction were expressed from the Xi, similar to an autosome, in these somatic cell hybrids, despite the ongoing inactivation of the majority of X-linked genes [19].

### Retention of DNA Hypomethylation at X-linked Gene Promoters upon Induction of Xist/XIST

**Figure 3:**
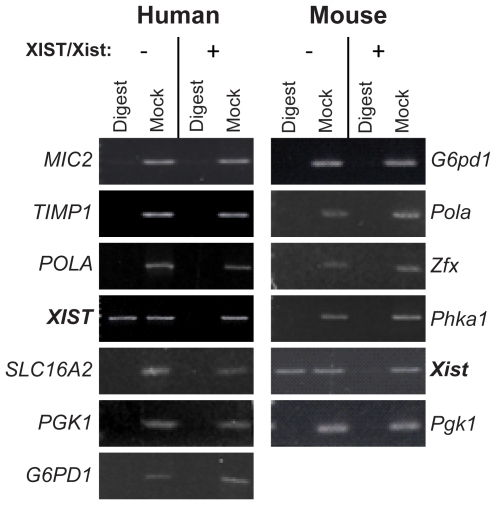
DNA methylation of X-linked gene promoters before and after expression of *XIST/Xist*. Genomic DNA was cut with a methylation-sensitive restriction enzyme prior to amplification by PCR with primers flanking the enzyme cut sites (lanes labeled Digest). Mock digested samples were used as a positive control (Mock). If a band occurs in the digest lane, it means that the site/sites within the amplicon are methylated (e.g. XIST/Xist). If no band was observed after PCR then we conclude that there was no intact template to amplify as the internal restriction enzyme sites were not methylated.

To analyze the methylation status of X-linked genes in the hybrid cells, we digested genomic DNA with a methylation-sensitive restriction enzyme followed by PCR with primers designed to flank three or more recognition sites. If any site within the amplicon was unmethylated, the enzyme would cut the template and there would not be a resultant PCR product. However, if the sites were methylated, the enzyme would not be able to cut the template and a band would be observed. Methylation analysis of 11 X-linked gene promoters showed that all the genes were hypomethylated in the cell line prior to *XIST/Xist* induction (AHA-11aB1) and continued to be hypomethylated in the hybrid expressing *XIST/Xist* (AHA-4C1) (Figure 3). Both the *XIST/Xist* genes demonstrated a change in promoter methylation that corresponded to their expression state. In the hybrid not expressing *XIST/Xist* (AHA-11aB1), both *XIST/Xist* gene promoters were methylated and in the hybrid expressing *XIST/Xist* (AHA-4C1) both promoters were not methylated. Therefore, the DNA methylation state at gene promoters is concordant with gene expression and *XIST/Xist* expression alone did not initiate DNA methylation changes at the promoters of X-linked genes in the somatic cell hybrids.

### Retention of Active Histone Modifications at X-linked Gene Promoters

**Figure 4:**
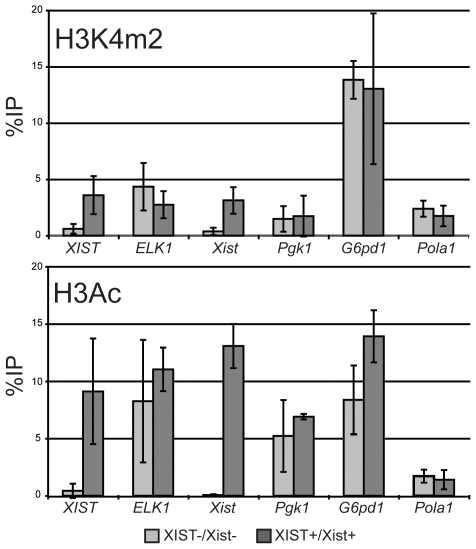
Active histone modifications at the promoters of X-linked genes after expression of *XIST/Xist*. H3K4m2 (upper) and H3Ac (lower) marks were assessed by ChIP followed by quantitative PCR for the two human and four mouse genes listed. The %IP is relative to input and error bars indicate error between 2–3 replicate immunoprecipitations.

To further assess whether active chromatin can be maintained in the presence of XIST/Xist we examined the promoter regions of X-linked genes for the active chromatin modifications H3K4 dimethylation (H3K4me2) and H3 acetylation (H3Ac) using chromatin immunoprecipitation (ChIP) followed by quantitative PCR. The only genes that changed expression as a result of the demethylation treatment were the *XIST/Xist* genes; they were not expressed in the AHA-11aB1 hybrid and were expressed in the AHA-4C1 hybrid (Figure 1D). After expression of *XIST/Xist* in the AHA-4C1 hybrid cell line, a gain of H3K4me2 and H3Ac was observed at both *XIST/Xist* promoter regions (Figure 4). Since human XIST was not localized to the human X chromosome while mouse Xist was localized, we focused on mouse genes as these were the most likely to be affected by *Xist* activation. None of the mouse X-linked genes, or the human *ELK1* gene, showed any significant decrease of these active marks as a result of *XIST/Xist* expression (Figure 4). Therefore these active chromatin marks are retained at the promoter regions of X-linked genes despite expression and localization of the Xist RNA.

## Discussion

The XIST/Xist non-coding RNA is critical for X inactivation, but it is unknown how the RNA localizes in *cis* and initiates silencing of the ∼150 Mb X chromosome. In order to examine which features associated with the Xi might result directly from XIST expression rather than the developmentally-associated silencing we created and analyzed a somatic cell hybrid (AHA-4C1) that stably expresses *XIST/Xist* from the active human and mouse X chromosomes. Previous analyses of somatic cell hybrids with induced *XIST/Xist* expression had shown a failure to induce X inactivation [12], [15], [16]. As the parental mouse cells have an active X chromosome it was anticipated that silencing of the human X would not be deleterious; however the induced human XIST failed to localize to the X chromosome, and thus it was not surprising that no silencing of human X-linked genes was observed. Failure of human XIST to localize is not a result of the demethylation treatement as inactive X chromosomes isolated in mouse/human somatic cell hybrids also fail to localize XIST ([12] and Figure 2G). Furthermore, Hansen et al. have shown that in human cells XIST induced by demethylation localizes, although the frequency of *XIST* reactivation is lower than in hybrid cells [16].

The failure of a transiently-induced *Xist* gene to induce silencing in these hybrid cells [12] as well as previous findings of a limited window of opportunity during development when Xist-mediated silencing could occur [6], [20] suggested that isolation of a stable *Xist*-expressing clone by demethylation would provide a cell line in which localized Xist expression and silencing are disconnected. If silencing of the sole mouse X chromosome did occur, it is possible that the presence of the human X might compensate given the conservation of X-linked genes across mammals [21]; however selection might also have occurred to generate a silencing defective clone. There may be rare genes that have in fact been silenced or partially silenced in these hybrids; however, the quantitative expression results showed that although all X-linked genes examined remained active, the demethylation treatment caused considerable changes in gene expression, suggesting that the demethylated hybrids are not an accurate model system for gene expression studies.

We have used these cells in which silencing and XIST/Xist expression are no longer connected to examine active chromatin marks, the loss of which are amongst the earliest known events following *Xist* expression during X inactivation in early development (reviewed in [1], [22]). We found that the human *XIST* and mouse *Xist* promoters gained H3Ac and H3K4m2 upon demethylation-induced expression; while the other X-linked promoters that we examined retained both marks. Therefore in these somatic cell hybrids *Xist* expression without silencing does not disrupt these active chromatin modifications. We also examined the recruitment of DNA methylation at X-linked promoters, which is generally considered to be a late event after the initial inactivation [23]. All the X-linked gene promoters we examined were hypomethylated in the hybrid cell line expressing *XIST/Xist*. Therefore the demethylation treatment and the resultant *XIST/Xist* expression did not cause these X-linked genes to become hypermethylated, rather the methylation state of these genes corresponded to their expression status.

The recruitment of repressive chromatin marks by the Xist RNA in the absence of silencing has been examined in several studies utilizing the Xist-delSX transgene that lacks the 5′ A-repeats and fails to induce transcription silencing during ES cell differentiation, despite apparently normal localization [2], [6], [7], [8], [9], [10], [11]. These studies have shown that *Xist* expression, in the absence of silencing was able to recruit H3K27m3 [7], [9], H4K20m1 [9], and macroH2A1[6]. It is important to note that compared to controls, the degree to which these events occurred with a silencing-impaired Xist RNA was varied, and that expression of *Xist* early in development, even when subsequently silenced, may contribute to later recruitment of chromatin marks [9]. In contrast, with our somatic hybrid system there was no early developmental expression of *Xist*; however, *Xist* is expressed from its endogenous promoter and expression levels are comparable to those of the BMSL2 female mouse cell line.

We assessed the presence of Cot-1 hybridization in our *Xist/XIST* reactivated somatic cell hybrids. Cot-1 DNA contains the middle repetitive fraction of genomic DNA and when used as a probe for RNA FISH, a depleted region, termed the Cot-1 hole, is associated with the Xi [2], [3], [17]. It was initially thought that this lack of hybridization reflected the global transcriptional repression of X-linked genes but recent work suggests that the Cot-1 hole represents a repressive nuclear compartment that can be established in the absence of genic silencing [2], [3]. Consistent with the ability to observe holes, we have shown that human chromosomes retained in mouse mouse/human somatic cell hybrids form transcriptional domains. These were observed for human autosomes and both the human Xa and Xi. The human Cot-1 domain associated with the human Xa in the AHA-11aB1 hybrid cells was not substantially altered by the reactivation of the *XIST* locus (generating the AHA-4C1 hybrid cells), suggesting that in addition to not silencing X-linked genes, the dispersed human XIST was not able to silence repetitive sequences. A human Cot-1 transcriptional domain was also associated with the Xi in a hybrid cell; therefore, the silent nature of the Xi did not impact the expression of the Cot-1 fraction. This supports the observation that the lack of Cot-1 RNA hybridization is not representative of genic silencing but instead reflects a repressive nuclear compartment that predominantly associates with repetitive non-genic silencing [2], [3].

In our Xa hybrid cells where mouse Xist is able to localize but not induce gene silencing (AHA-4C1), we observed substantially fewer Cot-1 holes (7%) as compared to a control cell line (BMSL2 - 59%) or to the frequencies reported during mouse development or ES cell differentiation [2], [4]. With the Xist-deltaSX transgene described above, silencing-deficient Xist expressed during ES differentiation formed a Cot-1 hole in the absence of gene silencing [2]. This could suggest that a developmental context improves the ability to establish a silent nuclear compartment. While Cot-1 holes have been reported upon XIST expression in human somatic cells [17], [24], these studies utilized transformed human cell lines, which may partially mimic the early developmental context. Furthermore, when an established Xi is introduced into a somatic cell hybrid, even with the maintenance of gene silencing, transcription of repetitive elements was visualized as a Cot-1 domain. This may reflect failure of repetitive element silencing in the absence of a localized XIST RNA signal, or failure to maintain repression of human repetitive elements in the somatic cell hybrid background. It has previously been shown that *XIST* expression is not required for maintenance of gene silencing [25], [26] and given our results it would be of interest to determine if repeat silencing was lost in the absence of XIST in human cells.

Induction of stable XIST/Xist required multiple rounds of treatment with the demethylating agent 5-aza, and thus it is not surprising that effects on loci other than *XIST* were observed. The hybridization of Cot-1 generally yields a diffuse staining pattern throughout the nucleus, however, very brightly stained mCot-1 regions were observed in our demethylated hybrid cells. These were not dependent upon induction of *XIST/Xist*, but rather seem to reflect other effects of demethylation. In addition we observed a decrease in *mPgk1* and *Uba1* expression from hybrids in which *Xist* had not been induced, consistent with previous studies which have reported differential gene expression resulting from 5-aza treatment [27], [28].

In the present study we have shown that stable *XIST/Xist* expression from its native locus, outside a developmental context, led to proper localization of mouse but not human XIST/Xist; however, X-linked genes remained active despite *Xist* expression and regardless of proximity to the *Xist* locus. *Xist* expression in the absence of silencing does not reduce H3K4m2 or H3Ac at promoter regions or cause a gain of DNA methylation. Our results support the view that the Cot-1 hole associated with the Xi need not be an indicator of gene silencing. The reduced ability to form a Cot-1 hole implicate the contribution of a developmental context or other specific chromatin modifications of the Xi for the establishment of a repressive nuclear compartment marked by the absence of Cot-1.

## Materials and Methods

### Cell Culture

The active X hybrid cell lines were grown in MEM supplemented with 7.5% fetal calf serum and penicillin/streptomycin at 37°C. The mouse parental line for the AHA-11aB1 Xa-containing hybrid was A9 [14], a heteroploid L-cell derivative previously reported to have a single copy of the X which has been involved in interchromosomal translocations [29]. AHA-4C1 (XIST+/Xist+) was derived from AHA-A5-2B (XIST+/Xist-) following treatment with 0.2–1.0 ug/ml 5-azacytidine and selection of *Xist*-expressing subclones as identified with RT-PCR using primers that span a splice junction (see supplementary Table S1 for primers). The inactive X hybrid cell lines were grown similarly but at 39°C to select for retention of the human Xi [14]. The female mouse fibroblast cell line, BMSL-2, was previously shown to retain an Xi, and grown in DMEM with 10% fetal calf serum and penicillin/streptomycin at 37°C [30]. GM04626 was grown in MEM supplemented with 20% fetal calf serum, penicillin/streptomycin and non-essential amino acids at 37°C.

### RNA Extraction & RT-PCR

RNA was extracted from cell pellets using Trizol (Invitrogen) following the manufacturer's protocol. Reverse transcription was performed with M-MLV (Invitrogen) and random hexamers (Pharmacia) for 2.5 hours at 42°C followed by PCR with primers from Table S1. Quantitative PCR was performed with cDNA as a template that was generated from reverse transcription with SYBR Green (Sigma) to quantify relative levels of cDNA.

### RNA-FISH

RNA-FISH was performed as previously described [17], [31]. Briefly, cells were grown on glass coverslips, permeablized with 0.5% Triton X-100 (Roche) and fixed in 4% paraformaldehyde. To detect human XIST, a DNA probe G1A, encompassing approximately 10 kb of genomic DNA extending from the fourth intron to the 3′ end of the *XIST* gene, was used [24]. To detect mouse Xist, a DNA probe of mouse Xist exon 1 was used [32] and to detect expression of Cot-1, Cot-1 DNA (Invitrogen, Human Cot-1 DNA #15279-011, Mouse Cot-1 DNA #18440-016) was used. The DNA probes were either labeled with the BioNick™ Labeling System (Invitrogen) and biotin-11dUTP (Roche) or digoxigenin-16-UTP (Roche), or with the Nick Translation Reagent Kit (Abbott Molecular Inc) and Spectrum red-UTP (Vysis), or Spectrum green-UTP (Vysis). The human and mouse probes would not distinguish *Xist/XIST* from *Tsix/TSIX*; however our PCR primers (see Table S1) are from the *XIST*-specific exon 1 for human and span an *Xist*-specific splice junction for mouse, suggesting that we are observing *Xist/XIST* RNA.

### Line Plot Analysis

A merged image was opened in ImageJ (NIH) and a line was drawn through the Xist/XIST signal. The image was then split into individual colours and the plot profile data was collected for each signal. The data was imported into an Excel (Microsoft) spreadsheet where the pixel intensities were calculated as deviation from the mean across the line and were plotted on a single graph.

### DNA Methylation

DNA was isolated from cultured cells and a PCR-based analysis of DNA methylation was performed as previously described [15]. Briefly, *Eco*RI digested DNA was additionally digested with *Hha*I (New England Biolabs) or *Hpa*II (New England Biolabs) and amplified with primers flanking the restriction enzyme sites within the promoter of the specific gene (see Table S1 for primers, enzymes used and number of restriction enzyme sites present within the amplicons). Mock-digested DNA was digested only with EcoRI for use as a positive-control for the PCR.

### ChIP

ChIP was performed as outlined by Upstate (Millipore). Briefly, the cells were cross-linked with 1% formaldehyde for 10 min at 37°C and quenched with 125 mM glycine. The cells were lysed and chromatin was sonicated to yield DNA sized between 200–1000 bp. The sonicated fraction was precleared with salmon sperm DNA/protein A agarose- 50% slurry (Upstate) and then incubated overnight with the antibody (anti-H3K4me2 (Upstate #07-030) and anti-H3Ac (Upstate #06-599)). The antibody/histone complex was collected with salmon sperm DNA/protein A agarose- 50% slurry and then the cross-links were reversed and the DNA was purified prior to quantitative PCR.

## Supporting Information

Table S1 Primer Table.
(0.10 MB DOC)
