# Supplementary material for: Active Chromatin Marks Are Retained on X Chromosomes Lacking Gene or Repeat Silencing Despite XIST/Xist Expression in Somatic Cell Hybrids
Source: PLoS One. 2010 May 24;5(5):e10787. doi: 10.1371/journal.pone.0010787 (PMC2875404; doi:10.1371/journal.pone.0010787)
Supplement: Table S1 — Primer Table. (0.10 MB DOC) [file pone.0010787.s001.doc]

Table S1: Primer Table

| **Gene** | **Product Size** | **Sequence** | **Anneal Temperature** |  |
| --- | --- | --- | --- | --- |
| **Expression Primers:** |  |  |  |  |
| mβActin | 123-130 | ctg gct cct agc acc atg aag atc | 59°C |  |
|  |  | tgc tga tcc aca tct gct gg |  |  |
| [[1]](#endnote-2)mAtrx | 316 | ggg att gct gct gtg agt ct | 59 |  |
|  |  | cca cca tct tct tgc cat ct |  |  |
| [[2]](#endnote-3)mChic1 | F-R: 308 | gag tgc cct tcc taa taa gtg g | 54 |  |
|  |  | ctg gaa ctc ata ctg tag acc agg |  |  |
|  | F-NR: 277 | tga act caa aga gat ctg tgg c |  |  |
| mPdha1 | 203 | gag cta aag gcg gat cag ctg tat | 59 |  |
|  |  | ctt cgt cct gtt agc tct gca aga |  |  |
| mPgk1 | 238 | aag cgc acg tct gcc gcg ctg ttc t | 59 |  |
|  |  | gtt ggc tcc att gtc caa gca gaa t |  |  |
| mPhka | 223 | aca cct gca gtt gga tgc tac ttc | 54 |  |
|  |  | ggc cat tcc aac tga act cgc att |  |  |
| ^1^mRnf12 | 378 | gag ccc cga tga aaa tag agc | 59 |  |
|  |  | ggt cgg cac ttc tgt tac tgc |  |  |
| mUba1 | 196 | agc tgt gct gca acg atg aa | 59 |  |
|  |  | gtc ttg agg ttg ctg ggt a |  |  |
| mZfx | 504 | cag ttg tca tcc agg atg tc | 59 |  |
|  |  | tcg ttg tcc ata gtc agt cc |  |  |
| ^2^mAbcb7 | 290 | aag cat tcg gca gtt ctg acc | 54 |  |
|  |  | tct agt atc aac atc ctt taa ccc |  |  |
| mXist | 578 | act gcc agc agc cta tac ag | 56 |  |
|  |  | gtt gat cct cgg gtc att ta |  |  |
| IDS | 300 | taa ggga gct gac tga tct tg | 54 |  |
|  |  | gct ata cgg aga atc atc g |  |  |
| XIST | 265 | cct ata ctg ctt aaa tgc gc | 54 |  |
|  |  | cct aag att atg cac gct aa |  |  |
| SLC16A2 | 155 | ttc aag gca tta acc tca ag | 54 |  |
|  |  | ggg ctc acc ata tca ata act |  |  |
| PGK1 | 395 | tcg gct ccc tcg ttg acc ga | 54 |  |
|  |  | agc tgg gtt ggc aca ggc tt |  |  |
| PLS3 | 210 | tga cct tgt gaa gag tgg c | 54 |  |
|  |  | acc tgc gaa tca tgc acc t |  |  |
| TAF1 | 220 | acc aag tgg cgt ttt ctt tc | 54 |  |
|  |  | gaa taa ggt tta cat cat cc |  |  |
| TIMP1 | 180 | aga tcc agc gcc cag aga ga | 56 |  |
|  |  | ccc tga tga cga ggt cgg aa |  |  |
| PDHA1 | 200 | cct gtg cgt ccg aga cgc | 54 |  |
|  |  | gtt cac cat cct gtc ctt g |  |  |
| **Methylation Primers:** |  |  |  | **Enzymes** |
| MIC2 | 373 | aga ggt gcg tcc gat tct t | 52 | 3 HpaII, 4 HhaI, 5 AciI |
|  |  | cgc cgc aga tgg aca att t | 1.0mM MgCl^2^, 4% DMSO |  |
| [[3]](#endnote-4)POLA | 348 | ctg ggg aaa acg atc caa cc | 60 | 5 HpaII |
|  |  | ctg aaa gcc aat cag cgg c | 1M betaine |  |
| TIMP1 | 294 | ccc ttg gct tct gca ctg a | 58 | 2 HpaII |
|  |  | cca agc tga gta gac agg c | 1.0mM MgCl^2^ |  |
| XIST | 555 | atg ctc tct ccg ccc tca | 54 | 3 AciI |
|  |  | Atc agc agg tat ccg ata cc |  |  |
| ^2^SLC16A2 | 129 | ctg gcc cgg ctc ctg gc | 62 | 8 HpaII |
|  |  | gct ttg ttt gcg cca acc tg | 2M betaine |  |
| ^2^PGK1 | 145 | acg cgg ctg ctc tgg gc | 62 | 3 HpaII |
|  |  | tta ggg gcg gag cag gaa g | 2M betaine |  |
| G6PD1 | 425 | cac tac gcg gag ctg cac | 54 | 7 HpaII |
|  |  | ctg aag cac aac aaa cag cgt | 1M betaine, 0.5mM MgCl^2^ |  |
| mXist | 392 | ttc tcg agc cag tta cgc ca | 58 | 4 HhaI |
|  |  | cca ttg cta cac acc aga ac |  |  |
| mSlc16a2 | 321 | cct gaa ctg tgt tct gcg t | 54 | 6 HpaII |
|  |  | agc ctg gaa ctt aga cac c | 1M betaine, 2mM MgCl^2^ |  |
| mPgk1 | 181 | ctt gag ggc agc agt acg gaa | 54 | 2 HpaII |
|  |  | ccg gca ttc tgc acg ctt caa |  |  |
| mZfx | 238 | ctc gtg cgg att tta cag c | 54 | 4 HpaII |
|  |  | agg aaa atg cgg aag ggt ag | 1M betaine |  |
| mPhka1 | 400 | cgt tca gtc cca gtc tct cag | 54 | 6 HpaII |
|  |  | aag acc ccg tct cca ctc a | 1M betaine |  |
| mPola | 257 | cat gcg tcc tac gga ttg tt | 54 | 3 HpaII |
|  |  | gaa agc caa tca gcg gcc t | 1M betaine |  |
| **ChIP Primers:** |  |  |  |  |
| XIST | 217 | gaa cca acc aaa tca cag aga | 58 |  |
|  |  | ata aag ggt gtt ggg gga c |  |  |
| ELK1 | 300 | gca cag ctc tgt agg gaa | 54 |  |
|  |  | agc tca cct gtg tgt agc g |  |  |
| mXist | 392 | ttc tcg agc cag tta cgc ca | 58 |  |
|  |  | cca ttg cta cac acc aga ac |  |  |
| mPgk1 | 181 | ctt gag ggc agc agt acg gaa | 54 |  |
|  |  | ccg gca ttc tgc acg ctt caa |  |  |
| mPola | 257 | cat gcg tcc tac gga ttg tt | 54 |  |
|  |  | gaa agc caa tca gcg gcc t |  |  |
| mG6pd1 | 214 | gcc cat gag gac tag acc tt | 54 |  |
|  |  | aca tcc act gtg ggc agc ta |  |  |

1. Kalantry S, Purushothaman S, Bowen RB, Starmer J, Magnuson T (2009) Evidence of Xist RNA-independent initiation of mouse imprinted X-chromosome inactivation. Nature 460: 647-651. [↑](#endnote-ref-2)
2. Huynh KD, Lee JT (2003) Inheritance of a pre-inactivated paternal X chromosome in early mouse embryos. Nature 426: 857-862. [↑](#endnote-ref-3)
3. Gilbert SL, Sharp PA (1999) Promoter-specific hypoacetylation of X-inactivated genes. Proc Natl Acad Sci, USA 96: 13825-13830. [↑](#endnote-ref-4)
